# Factors that contribute to public sector nurses’ turnover in Limpopo province of South Africa

**DOI:** 10.4102/phcfm.v5i1.479

**Published:** 2013-07-29

**Authors:** Takalani G. Tshitangano

**Affiliations:** 1 Department of Public Health, University of Venda, South Africa

## Abstract

**Background:**

The ongoing worldwide phenomenon of a shortage of about 4.3 million nurses and midwives poses a threat to health service delivery. Limpopo province had the worst nurse shortage of over 60% in 2010. Authors attribute this shortage to turnover of nurses. The quest to describe factors contributing to nurses’ turnover led to this study in Limpopo province, South Africa.

**Objectives:**

To explore and describe factors that contribute to nurses’ turnover in Limpopo province of South Africa by assessing public sector nurses’ job satisfaction in relation to common determinants of job satisfaction.

**Method:**

A descriptive cross-sectional approach used primary quantitative data collected from 141 of 380 respondents (31.1% response rate) contacted incidentally. Self-administered hand-delivered questionnaires were used to gather ordinal data, which were analysed in terms of frequency and percentage tables using the Statistical Package for Social Sciences version 6. The sum of positive and negative effects was used to determine satisfaction; if positive effects were greater than negative effects respondents were judged to be satisfied and vice versa.

**Results:**

Frequency and percentage tables revealed that nurses in Limpopo province were more dissatisfied (53.9%) than satisfied (37.8%) with their jobs. Factors which respondents were found to be dissatisfied with included staffing (85.2%), availability of workplace resources (83.7%), salaries (78.8%), workplace safety (73.7%), career development opportunities (64.5%) and hours of work (47.6%).

**Conclusion:**

Nurses’ turnover is attributed to nurses’ dissatisfaction with staffing, resources, salaries and workplace safety. Attention needs to be given to these specific issues if retention of nurses is to be achieved.

## Abstrait

**Les facteurs qui contribuent à la rotation des infirmiers du secteur public dans la province du Limpopo en Afrique du Sud**

**Contexte:**

Le phénomène mondial actuel de pénurie d'environ 4,3 millions d'infirmiers et de sages-femmes fait peser une menace sur l'offre de services de santé. La province du Limpopo a connu la plus grave pénurie en infirmiers en 2010, à plus de 60%. Les auteurs attribuent cette pénurie à la rotation des infirmiers. La quête visant à décrire les facteurs contribuant à la rotation des infirmiers a mené à cette étude dans la province du Limpopo, en Afrique du Sud.

**Objectifs:**

Explorer et décrire les facteurs qui contribuent à la rotation des infirmiers dans la province sud-africaine du Limpopo en évaluant la satisfaction professionnelle des infirmiers du secteur public en relation avec des déterminants courants de satisfaction professionnelle.

**Méthode:**

Une approche transversale descriptive a été employée en utilisant les données quantitatives recueillies auprès de 141 des 380 sondés (taux de réponse de 31,1%) contactés à ce propos. Des questionnaires remis en main propre et auto-administrés ont été utilisés pour rassembler des données ordinales, qui ont été analysées selon des tableaux de fréquence et de pourcentage en utilisant le logiciel « Statistical Package for Social Sciences », version 6. La somme des effets positifs et négatifs a été utilisée pour déterminer la satisfaction; si les effets positifs étaient supérieurs aux effets négatifs, les sondés étaient considérés comme étant satisfaits et vice-versa.

**Résultats:**

Les tableaux de fréquence et de pourcentages ont indiqué que les infirmiers dans la province du Limpopo étaient plus mécontents (53,9%) que contents (37,8%) de leur travail. Les facteurs, dont les sondés étaient mécontents, incluaient la dotation en personnel (85,2%), la disponibilité de ressources sur le lieu de travail (83,7%), les salaires (78,8%), la sécurité sur le lieu de travail (73,7%), les opportunités d'avancement professionnel (64,5%) et les heures de travail (47,6%).

**Conclusion:**

La rotation des infirmiers est attribuée au mécontentement des infirmiers quant à la dotation en personnel, aux ressources, aux salaires et à la sécurité sur le lieu de travail. Une attention doit être accordée à ces problèmes spécifiques si l'on veut fidéliser les infirmiers.

## Introduction

There is an ongoing worldwide phenomenon of a shortage of nurses. The World Health Organization (WHO) estimated that there was a global shortage of about 4.3 million nurses and midwives in 2010.^1^ South Africa had 32 000 vacant registered nurses’ posts in 2010,^2^ whilst it was estimated that the country will have a shortage of 20 815 nurses in 2015.^3^ The Health Systems Trust^4^ revealed shocking figures for registered nurses shortages in 2011, which indicated that Limpopo province had the worst nurse shortage, of above 60%. Nursing shortages can be divided into those caused by recruitment difficulties and/or retention difficulties.^5^ Shields and Ward^6^ as well as Saari and Judge^7^ stated that working conditions are the major causes of retention difficulties as they determine job satisfaction.

### Problem statement

Despite efforts by the South African National Department of Health to recruit and retain staff, including nurses’ occupation-specific dispensation (OSD) and retention policies, about 2000 South African nurses were lost to the United Kingdom (UK) in 2001–2002 and about 300 more were leaving South Africa every month.^8^ According to the South African Nursing Council (SANC) register^9^ more than 18% of nurses no longer practice as nurses. Another 18% of these nurses are registered with nursing agencies as depending solely on moonlighting in private hospitals, whilst others are permanently employed in private hospitals.^9^

South Africa is amongst the three countries with the highest percentages of nurses who intend to migrate, aggravating the nursing shortages in the country.^10^ Even though Aiken^11^ advocates that the optimal workload of one nurse is four patients, in rural areas provinces like Limpopo still have an average of one nurse responsible for 40 patients on night shift. On afternoon shift one nurse was responsible for an average of 25 patients.^12^ Even though the country exceeds the WHO's absolute minimum nurse staffing norm/standard of 200 nurses per 10 000 individuals (which equals 500 people per one nurse), some of its provinces, like Limpopo, Mpumalanga and the Northern Cape, had the worst staffing ratios (of 732:1, 662:1 and 583:1 respectively).^13^

The shortage of qualified nurses in South Africa is highlighted as a barrier to achieving the goals of the Department of Health to make health care services accessible, affordable, equitable and acceptable.^14, 15^

#### Study question

What are the factors contributing to public sector nurses’ turnover in the Limpopo province of South Africa?

#### Purpose of the study

This study aimed to explore and describe factors contributing to turnover of public sector nurses in the Limpopo province of South Africa, by examining their job satisfaction with regard to 12 common determinants of job satisfaction.

#### Objectives

This study aimed to accomplish the following objectives:

- To assess public sector nurses’ job satisfaction with regard to 12 common determinants of job satisfaction.
- To evaluate the rate of nurses’ job satisfaction.
- To evaluate the rate of nurses’ job dissatisfaction.

#### Rationale

Uncountable numbers of studies have been conducted overseas and in South Africa to assess job satisfaction of employees, including nurses. However, no such studies have been conducted in Limpopo province. The study findings will add to the body of existing job satisfaction research worldwide, and initiate a job satisfaction data base for Limpopo province in South Africa.

#### Contribution to field

The study's findings will inform bargaining council negotiations in relation to conditions of service, including salary and hours of work. Hospital managers will know what to emphasise during management meetings with regard to workplace relations. If the recommendations of this study are implemented, the job satisfaction of public sector nurses will improve. It is believed that improved job satisfaction will increase the retention rate and reduce the turnover of public sector nurses.^16, 17, 18^

## Research method and design

### Approach and design

This study adopted a quantitative approach using a cross-sectional descriptive survey design to explore and describe factors that contributed to nurses’ turnover in public health institutions (hospitals, health centres and clinics) around Limpopo province.

### Setting

Limpopo province is situated in the north-eastern corner of the Republic of South Africa. This province shares international borders with three other countries: Botswana to the west and north-west, Zimbabwe to the north, and Mozambique to the east. The Limpopo Department of Health has 49 hospitals, 27 health centres and 408 clinics.^18^

### Target population

All registered professional nurses in the public health facilities of Limpopo province were eligible for the study, whilst staff nurses, assistant nurses and student nurses were excluded. According to the SANC register^13^ there were 7743 registered nurses in Limpopo province in 2010.

### Sampling method

The difficulties of obtaining a list of all registered nurses from the Limpopo Provincial Department of Health as well as from the SANC compelled the researcher to use convenience sampling.

#### Sample size

The sample size of registered nurses who participated in this study was calculated using the following Solvin's formula, where N is the total number of number of registered nurses in Limpopo province, n is the sample size and e is the accepted level of error of 0.05:

*N* = N

1+ (N x e2) (Burns & Grove, 200719).

There were about 7743 registered professional nurses in Limpopo Province^13^:

*n* = 7743

 1 + (7743 X 0.0025)

*n* = 7743

 1 + 19.3575

*n* = 7743

20.4

*n* = 380

Thus, the sample size of this study was 380 registered nurses.

### Data collection method and instrument

A self-administered questionnaire was the data collection method in this study. The researcher designed the questionnaire in the form of a five-point Likert scale. When responding to this Likert scale survey instrument respondents were asked how strongly they agreed or disagreed with each statement, with numeric values allocated to the responses, such as Strongly agree (4), Agree (3), Disagree (2), Strongly disagree (1), and I do not want to answer (0).

Questions determined nurses’ satisfaction with each of 12 common determinants of job satisfaction, namely: quality of care, autonomy, clinical supervision, professional interpersonal relationships, salary, workplace safety, staffing, career development opportunities, hours of work, mission and purposes of the organisation, and availability of workplace resources. A person's attitude was measured by combining the responses across all items.^20^

#### Data collection process

Registered nurses were approached, recruited and given questionnaires as they were met during tea or lunch breaks outside hospitals, health centres and clinics, until all 380 questionnaires were distributed. Only 191 (47.75%) questionnaires were returned, of which 141 (35.2%) were correctly filled in. Thus, only 141 questionnaires were analysed.

### Analyses

Data were analysed by means of frequencies and percentages using the Statistical Package for Social Sciences (SPSS), version 6. The sum of positive and negative effects as evidenced by these results was used to determine the job satisfaction of nurses. If the positive effects were greater than the negative effects, respondents were judged to be satisfied and vice-versa.

## Results and discussion

Limpopo province is mostly rural and hence has health institutions in rural areas; the majority of respondents (93.6%) were working in rural areas. Most respondents were female nurses (79.4%), congruent with SANC statistics that nursing is a profession for women. In 2011 the SANC register had 109 332 females compared to 8930 males.^13^ Four nurses (2.8%) did not respond to this question.

The highest percentage of nurses (46.1%) was aged 36–45 years. According to Cowey and Gardiner^21^ the nursing generation born between 1960 and 1980 (now aged 27–47 years) is called generation X. This generation is said to be very difficult to retain in professions such as nursing since they lack job loyalty, have an excessive need for independence and are willing to change jobs. Furthermore, nurses in their twenties and thirties view the workplace differently, preferring greater autonomy and less bureaucracy. They choose independent work, such as working for temporary agencies in health care.^22^ Hence there is still going to be shortages as a result of turnover of this group.

In addition, turnover was found to be highest amongst nurses younger than 30 years of age.^6^ Many countries, like the UK, United States of America, Ireland, Canada, Australia and New Zealand, are using respondents’ age to predict future staff shortages.^11^ Thus one may speculate that by 2018 South Africa will lose only about 2.8% of nurses due to retirement, and 9.2% by 2028.

The findings of this study are in line with the age distribution of registered nurses obtainable from the SANC web site, which shows only 6% of registered nurses in South Africa to be less than 30 years of age; 23% between the ages of 30–39 years; 35% between 40–49 years; 25% between 50–59 years; and 11% between 60–69 years.^13^

The highest percentage (29.1%) of respondents was less experienced, with less than 4 years’ nursing experience. Less experienced nurses are new graduates,^23, 24, 25^ and new graduates belong to generation Y. According to Thoni^23^ generation Y members leave university or college properly armed with considerable knowledge, but lacking workplace experience. This may be a challenge to the quality of nursing care they render. Furthermore, generation Y members lack job loyalty and are unwilling to tolerate the previous rigid health care management system. Thus, a high turnover of nursing staff is possible in the foreseeable future.^26^

There were not many nurses with more than 5 years’ experience: 16.3% had 5–10 years’ nursing experience; 19.1% had 11–15 years; and 19.1% had 16–20 years. Nurses with more than 5 years’ nursing experience belong to generation X, which is characterised by willingness to change jobs; much of the nursing shortage is due to failure to retain this generation.^21^

Very few respondents (9.9%) were more experienced (over 20 years of nursing experience). The majority of respondents (84.4%) did not have any special nursing skills such as psychiatry, advanced midwifery, trauma nursing, intensive care nursing, nursing education, primary health care, etc. This may impact on the quality of care rendered by these nurses to patients. Only 13.5% of the respondents possessed various nursing specialties such as those mentioned above. Two respondents did not respond to this question.

Findings of this study on nurses’ job satisfaction are summarised in Table 1.

**TABLE 1:** Average job satisfaction scores of registered nurses in health care institutions of Limpopo province.

Job satisfaction determinant	Frequency	% satisfied	Frequency	% dissatisfied
Workplace resources availability	22	15.6	119	84.3
Autonomy	73	51.7	58	41.1
Professional interpersonal relationships	91	64.5	50	35.4
Clinical supervision	90	63.8	51	36.17
Workplace safety	37	26.2	104	73.7
Hours of work	68	48.2	73	51.7
Quality of care	86	60.9	55	39.0
Organisational mission	93	65.9	48	34.0
Salary	30	21.2	111	78.7
Career development opportunities	50	35.4	91	64.5
Staffing	25	17.7	116	82.2
Nursing work	88	62.4	53	37.58
Job satisfaction average = sum / 12	64	45.4	77	54.6

### Satisfaction with salary

The majority of respondents (78.7%) were dissatisfied with their salaries, similar to the findings of Minnaar, Reid and Naidoo,^25^ who revealed that nurses were most satisfied with the factor reflecting “personal satisfaction about their contribution to the work”. Furthermore, nurses were least satisfied with the factor to do with pay prospects. Similarly, it was found that pay-related issues and training opportunities dominated as the main problems at work.^27^

The data for this study were collected immediately after nurses had just received back payment of their salary scales adjustment in terms of the OSD.^28^ In terms of these results the OSD could only satisfy the salary expectations of 21.3% of nurses in rural areas.

These findings confirm those of previous studies which reported that registered nurses worldwide had the lowest satisfaction with pay, benefits or incentives.^29, 30, 31, 32^ In South Africa it has also been shown that pay-related issues dominated as the main problems at work^27, 33, 34^ As such it seems likely that the OSD did not make things any better in Limpopo province.

Individuals’ satisfaction has been reported to be affected by their absolute income level, or by their income as compared to others.^6^ Thus, much of the dissatisfaction amongst nurses might be due to individuals’ comparison of their present salary grade with the benchmark opportunities open to them, as well as comparing themselves with other public service workers^35, 36^ and their peers in better paid jobs.^37^ Furthermore, the largest determinant of overall job satisfaction is not being graded fairly in accordance with one's duties.^6^

Employees who are not satisfied with their pay are more likely not to perform to their full potential. Furthermore, some employees who are not satisfied with pay steal the organisation's resources, seeing such theft as a morally justifiable supplement to their wages.^37^ This could be a reason behind recent media reports in South Africa where nurses have been found in possession of public sector medication which was being sold for cash.^38^ Buchan^39^ argues that nurses support a workplace that provides equitable remuneration in a work system that is flexible and focused on providing quality patient care.

### Satisfaction with workplace safety

The majority of respondents (73.7%) strongly disagreed that they were satisfied with their safety at their workplace. Previous studies have reported that certain factors were associated with the public assaulting health care workers,^40^ and the employer considers assaults to be part of the job.^40^ The presence of security personnel in clinics in Limpopo province reduces the rate of assaults.

Stone *et al*.^41^ mentioned that health and safety concerns compel nurses to continue seeking the kind of nursing work they choose to perform. Similarly, Creegan, Duffield and Forrester^42^ reported that nurses leave the acute care setting looking for those with more sociable hours. Thus, concerns over safety hinder recruitment efforts and may contribute to hospital personnel shortages.

Furthermore, nurses are exposed to thousands of chemicals and other toxic substances in practice, which include aerosols, gases and skin contaminants from medications. This exposure results in health care workers contracting serious infections such as hepatitis B or C virus or HIV infection.^43, 44, 45, 46, 47, 48^

### Satisfaction with career development opportunities

The majority of respondents (64.5%) were dissatisfied with career development opportunities at their workplace. This is in line with the number of nurses with special nursing skills, mentioned above; only 19 out of 141 nurses having nursing specialties. Promotions upward or horizontally go hand in hand with training and staff development. If nurses are not trained, it means they may not be suitable for promotion.

These findings are congruent with previous studies that mentioned that lack of career development and professional status play a role in nurses’ job satisfaction.^36, 49^ Furthermore, Shields and Ward^6^ found dissatisfaction with promotion and training opportunities to have a stronger impact on nurses’ retention than salary.

### Satisfaction with organisational mission

The majority of respondents (65.9%) were satisfied with the mission and purposes of their institution. According to Herzberg and Mausner's^49^ theory, company policy and administration are regarded as ‘dissatisfaction avoidance or hygiene’ factors which need to be attended to in order to achieve employee satisfaction.

### Satisfaction with quality of care rendered

The majority (60.9%) were satisfied with the quality of care rendered in their institution. It is not clear whether this was by chance or whether it was true; Lussier^50^ discovered that job dissatisfaction was also derived from co-workers who do not provide good care.

### Satisfaction with hours of work

With regard to hours of work, there was no significant difference between the number of respondents who disagreed (51.7%) and agreed (48.2%) that they were satisfied with their hours of work. Shift patterns also have an important influence on overall job satisfaction.^6^ Those nurses working shifts such as night duty have lower job satisfaction levels than respondents working only during the day. Unsociable hours make work and family balance even more difficult to attain, and may well force a shift from nursing to an alternative career.^21^

### Satisfaction with clinical supervision

About 63.8% of respondents agreed that they were satisfied with clinical supervision. It has been revealed that the mentor's role in assisting nurses to reach professional excellence is very important in nurses’ retention. Lussier^50^ asserts that clinical mentors play an important role in increasing nurses’ self-confidence, promoting role socialisation and encouraging independence, which leads to clinical competency. Similarly, Begat and Severeinsson45 highlighted that clinical nurse specialists’ support has a positive effect on nurses’ perceptions of well-being.

### Satisfaction with professional interpersonal relationships

About 64.5% of respondents were satisfied with professional interpersonal relationships at work. Several studies reported that nurses were generally satisfied with the interaction they have with colleagues, but not with higher level management, hence the marginally higher satisfaction rate.^17, 36^ This is positive given that optimal health provision depends on teamwork and interprofessional cooperation and communication.^17, 36^

### Satisfaction with autonomy

With regard to autonomy, about half of the respondents (51.8%) were satisfied with the autonomy they were given at work. Autonomy is attributed to job satisfaction by many authors.^33, 43, 44, 45, 51^

### Satisfaction with workplace resource availability

The majority of respondents (84.3%) disagreed that they were satisfied with the availability of resources at their workplaces. This concurs with Pillay's^34^ finding that nurses received a lot of insults and mistrust from the public due to shortages of medicines at hospitals and clinics.

## Ethical considerations

Approval to conduct this study was granted by the Limpopo Provincial Department of Health. Each participant was given a leaflet containing information such as how their anonymity, confidentiality and privacy would be ensured, including their right to withdraw from the study at any time should they feel the need to do so. After the researcher was sure that participants were satisfied with the information given, they were asked to complete and sign consent forms which were handed over to the researcher at the point of data collection. Participants who signed and submitted consent forms were then issued with a study questionnaire, which they completed on their own in the presence of the researcher.

## Trustworthiness

### Reliability

A pilot study was conducted on 10% (38) individuals with the same characteristics as the study population by two different research assistants who administered the same questionnaires to them. The correlation coefficient of 0.67 matched the 0.70 which is considered acceptable for a newly developed instrument.^19^

### Validity

An industrial psychologist was consulted to verify the job satisfaction terminology used, relevancy of the content and whether the instrument covered all important aspects of job satisfaction, thus ensuring content-related validity.

## Limitations of the study

The study sampling technique was incidental, which does not guarantee fair representativity, hence limiting generalisability of the findings. The sample size was small, which further limits the generalisabilty of the study findings.

The findings of this study serve as a basis for future research which aims to determine the impact which each determinant has on job satisfaction and employee turnover. Job satisfaction has been proved to motivate job performance.^25^ Employers should always strive to satisfy nurses by improving their conditions of service – especially those determinants which nurses were found to be dissatisfied with – if quality nursing provision is to be realised in the Limpopo province of South Africa.

### 

#### Recommendations

It is believed that improved levels of job satisfaction could assist in reducing turnover rates amongst registered nurses in South Africa.^16, 17^ It is therefore recommended that public hospital managers in Limpopo province must look for ways to improve nurses’ satisfaction with each of the 12 determinants of job satisfaction as well as personal characteristics in order to enhance nurses’ job satisfaction and reduce turnover.

## Conclusion

Overall the results of this study reveal that nurses are more dissatisfied with their jobs, with 54.6% being dissatisfied compared to 44.5% who were satisfied. Six factors which respondents were dissatisfied with were staffing (82.2%), availability of workplace resources (84.3%), salaries (78.7%), workplace safety (73.7%), career development opportunities (64.5%), and hours of work (51.7%). The remaining six factors, which the public sector nurses were satisfied with, were autonomy (51.8%), nursing work itself (62.4%), professional interpersonal relationships (64.5%), clinical supervision (63.8%), and the mission of their organisation (65.9%).

It is therefore concluded that nurses’ turnover may be attributed to their dissatisfaction with the quality of care rendered in an institution; autonomy; type of supervision; professional interpersonal relationships; salary; workplace safety; staffing; career development opportunities; hours of work/shift work; mission and purposes of the institution; availability of workplace resources and location of the facility (rural or urban).
